# Amino Acid Deletions in p6^Gag^ Domain of HIV-1 CRF07_BC Ameliorate Galectin-3 Mediated Enhancement in Viral Budding

**DOI:** 10.3390/ijms21082910

**Published:** 2020-04-21

**Authors:** Wen-Hung Wang, Chun-Sheng Yeh, Chih-Yen Lin, Ruei-Yu Yuan, Aspiro Nayim Urbina, Po-Liang Lu, Yen-Hsu Chen, Yi-Ming Arthur Chen, Fu-Tong Liu, Sheng-Fan Wang

**Affiliations:** 1 Center for Tropical Medicine and Infectious Disease, Kaohsiung Medical University, Kaohsiung 80708, Taiwan; bole0918@gmail.com (W.-H.W.); pigpipi831205@gmail.com (C.-Y.L.); aspiro.urbina@hotmail.com (A.N.U.); d830166@kmu.edu.tw (P.-L.L.); d810070@kmu.edu.tw (Y.-H.C.); 2 Division of Infectious Disease, Department of Internal Medicine, Kaohsiung Medical University Hospital, Kaohsiung Medical University, Kaohsiung 80708, Taiwan; 3 Department of Medical Laboratory Science and Biotechnology, Kaohsiung Medical University, Kaohsiung 80708, Taiwan; yeh30562@gmail.com (C.-S.Y.); kenny9517532000@gmail.com (R.-Y.Y.); 4 Graduate Institute of Medicine, Kaohsiung Medical University, Kaohsiung 80708, Taiwan; arthur@kmu.edu.tw; 5 Master Program in Clinical Pharmacogenomics and Pharmacoproteomics, College of Pharmacy, Taipei Medical University, Taipei 110, Taiwan; 6 Institute of Biomedical Sciences, Academia Sinica 11529, Taiwan; ftliu@ibms.sinica.edu.tw; 7 Department of Medical Research, Kaohsiung Medical University Hospital, Kaohsiung Medical University, Kaohsiung 80708, Taiwan

**Keywords:** Galectin-3, HIV-1, CRF07_BC, p6^Gag^, Alix

## Abstract

HIV-1 CRF07_BC is a recombinant virus with amino acid (a.a.) deletions in p6^Gag^, which are overlapped with the Alix-binding domain. Galectin-3 (Gal3), a β-galactose binding lectin, has been reported to interact with Alix and regulate HIV-1 subtype B budding. This study aims to evaluate the role of Gal3 in HIV-1 CRF07_BC infection and the potential effect of a.a. deletions on Gal3-mediated regulation. A total of 38 HIV-1+ injecting drug users (IDUs) were enrolled in the study. Viral characterization and correlation of Gal3 were validated. CRF07_BC containing 7 a.a. deletions and wild-type in the p6^Gag^ (CRF07_BC-7d and -wt) were isolated and infectious clones were generated. Viral growth kinetic and budding assays using Jurkat-CCR5/Jurkat-CCR5-Gal3 cells infected with CRF07_BC were performed. Results indicate that 69.4% (25/38) of the recruited patients were identified as CRF07_BC, and CRF07_BC-7d was predominant. Slow disease progression and significantly higher plasma Gal3 were noted in CRF07_BC patients (*p* < 0.01). Results revealed that CRF07_BC infection resulted in Gal3 expression, which was induced by Tat. Growth dynamic and budding assays indicated that Gal3 expression in Jurkat-CCR5 cells significantly enhanced CRF07_BC-wt replication and budding (*p* < 0.05), while the promoting effect was ameliorated in CRF07_BC-7d. Co-immunoprecipitation found that deletions in the p6^Gag^ reduced Gal-3-mediated enhancement of the Alix–Gag interaction.

## 1. Introduction

HIV-1 CRF07_BC is a recombinant of the B′ (the Thailand variant of subtype B) and C subtypes of the HIV-1 virus. Previous studies reported that CRF07_BC originated in Yunnan, China, where subtype B′ from Thailand mixed with subtype C from India before moving to Xinjiang Province, along a major Chinese heroin trafficking route [1,2]. In 2004, HIV-1 CRF07_BC caused an outbreak in Taiwan [3,4,5]. The sequence alignment and comparison of the full-length genome of the Taiwanese CRF07_BC found natural deletions in the p6^Gag^ [3,6]. Recently, studies demonstrated that HIV-1 CRF07_BC infection displayed low immunological progression in patients compared to the patients infected with subtype B, however the mechanism is still not fully understood [7,8].

The HIV-1 replication cycle entails three phases, namely attachment and entry, gene and protein expression, and assembly and budding. Viral Gag proteins are known to play a key role in the assembly and release of HIV-1 [9,10]. During viral replication, the Pr55^gag^ polyprotein is translated and further cleaved into matrix protein (p17; MA), capsid protein (p24; CA), p2, nucleocapsid protein (P7; NC), p1, and p6^gag^. Around 5% Pr160^gag-pol^ polyprotein is driven from ribosomal shrift and cleaved into MA, CA, P2, NC, p6^pol^, protease (PR), reverse transcriptase (RT), and integrase (IN) [11,12]. HIV-1 p6^Gag^ has two late domains, Pro-Thr/Ser-Ala-Pro (PT/SAP) and Tyr-Pro-Xn-Leu (YPXnL), where “X” is a variable residue and “*n*” is 1–3 motifs that interact with Tsg101 (yeast Vps23p) and apoptosis-linked gene 2 (ALG-2)-interacting protein (Alix, formerly known as AIP1), respectively. Tsg101 and Alix both function at the endosome to help sort membrane proteins into vesicles that bud into the lumen to create multivesicular bodies (MVBs). Tsg101 and Alix are primarily associated with endosomal sorting complex required for transport (ESCRT). They are important for the recognition of modified membrane proteins due to ubiquitination and the sorting of cargoes into membrane domains for the formation of the intralumenal vesicles (ILVs) of multivesicular bodies [13]. Knockdown or blocking of the interaction between Tsg101 or Alix with p6^Gag^ were reported to ameliorate HIV viral assembly and budding [14,15,16].

Galectin-3 (Gal3), a member of the β-galactoside-binding lectin family, has a carbohydrate recognition-binding domain (CRD) containing an estimate of 130 amino acids (a.a.) linked to a non-lectin N-terminal region (approximately 120 amino acids), which causes lectin oligomerization and ligand cross-linking [17,18]. Galectin-3 has been reported to have multiple functions involved in cellular, physical, and immune regulations [19]. Galectin-3 has also been described as an element of intracellular vesicles, such as phagosomes or exosomes [20]. The functions of galectin-3 mainly rely on its interaction with surface glycoconjugates extracellularly and cellular components intracellularly via lectin–glycan and protein–protein interactions, respectively [18,21,22,23,24]. Furthermore, galectin-3 has been associated with Alix at the cytosolic phase of the immunological synapse of activated T cells [25]. 

To date, a few reports have studied the role of galectin-3 in virus infection [26]. Regarding HIV-1, Fogel et al. indicated that galectin-3 was upregulated in MOLT-3 T cells during HIV-1 subtype B post-infection [27]. Our previous work demonstrated that galectin-3 promoted HIV-1 budding via association with Alix and p6^Gag^ [28]. These findings may hint that galectin-3 is dependent on Alix to interact with Gag and further regulates HIV-1. Combined, it is known that (1) amino acid deletions have been detected in the p6^Gag^ of HIV-1 CRF07_BC, (2) the Alix–p6^Gag^ interaction plays an important role in HIV-1 assembly and budding, and (3) HIV-1 CRF07_BC infection displays slow disease progression [7,8]. We therefore hypothesized that galectin-3 plays a role in HIV-1 CRF07_BC infection and amino acid deletions in the p6^Gag^ may affect the regulation induced by galectin-3. Thus, this study aims to address the correlation of galectin-3 to CRF07_BC infection and the potential effect of amino acid deletions on the p6^Gag^ on galectin-3-mediated regulation. 

## 2. Results

### 2.1. Slow Disease Progression and Higher Plasma Galectin-3 Detected in HIV-1 CRF07_BC-Infected Patients 

**Figure 1:**
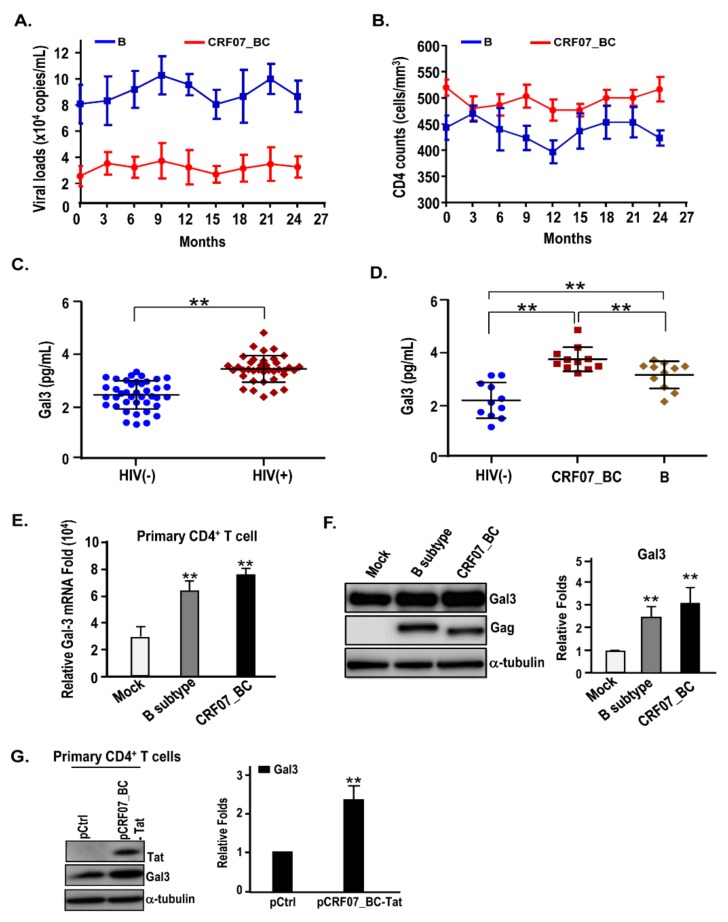
Slow disease progression and higher galectin-3 in plasma were detected in CRF07_BC-infected patients. The comparison of the disease progression between the patients infected with B subtype (*n* = 5) and CRF07_BC (*n* = 5) was conducted. Their (**A**) viral load and (**B**) CD4 counts were monitored. (**C**) The concentrations of galectin-3 in the plasma from healthy donor and HIV-1(+) patients were compared. (**D**) The concentrations of galectin-3 in the plasma from different genotypes of HIV-1-infected patients were compared. (**E**) Galectin-3 mRNA expression level in control and different genotypes of HIV-1-infected primary CD4^+^ cells were validated using qRT-PCR. (**F**) Galectin-3 protein expression levels in control and different genotypes of HIV-1-infected primary CD4^+^ cells were validated using immunoblotting. The intensities of the band were quantified by densitometry. The related fold was determined by the intensities of Gal3 normalized with the intensities of α-tubulin. (**G**) Evaluated galectin-3 expression in human primary CD4^+^ T cells via electroporation with control and CRF07_BC-Tat expressing vectors. Representative results are shown. Quantitative data represent the means ± standard deviation (SD) of results from at least three independent experiments (** *p* < 0.01).

Firstly, we addressed the characteristics of HIV-1 CRF07_BC and the effects of galectin-3 in HIV-1 CRF07_BC. A total of 38 HIV-1+ injecting drug user (IDU) patients with non-cART treatment signed the informed consent and were enrolled. The blood samples were collected and subjected to viral load and CD4 count measurement. Results indicated that 25 (65.7%) subjects had viral loads below 100,000 copies/mL and 32 (84.2%) subjects had CD4 counts above 200 cells/mm^3^ (Table 1). Data from HIV-1 genotyping determination indicated that 5 (13.2%), 8 (22.2%), and 25 (69.4%) subjects belonged to the CRF01_AE, B, and CRF07_BC subtypes, respectively (Table 1). The subtypes C and CRF08_BC were not found in our cohort (Table 1). Among CRF07_BC infections, the CRF07_BC carrying 7 amino acid deletion in the p6^Gag^ was predominant (23/25; 92%) (Table 2). Next, the disease progression of CRF07_BC-infected patients were validated. Results indicated that significantly lower viral load and higher CD4 count were found in the CRF07_BC-infected patients compared to the B subtype-infected patients during longitudinal observation (*p* < 0.01) (Figure 1A,B). We further measured the galectin-3 concentrations in the plasma samples and found that significantly higher galectin-3 was detected in HIV-1(+) patients than the HIV-1(-) control groups (*p* < 0.01). Furthermore, results also indicated that significantly higher galectin-3 was detected in patients infected with CRF07_BC than the patients infected with the B subtype (*p* < 0.01). 

### 2.2. HIV-1 CRF07_BC Infection Induced Galectin-3 Expression

Previous studies have indicated that HTLV-1 infection [29] and Tat of HIV-1 B subtype induce expression of galectin-3 [27]. Another study reported that Tat of HIV-1 CRF07_BC was B’/C recombinant, with major B’ subtype linked with the C subtype [30]. Accordingly, we hypothesized that Tat protein of CRF07_BC may have a similar induction effect to trigger galectin-3 expression. The primary CD4^+^ cells from healthy donors were exposed to HIV-1 subtype B NL4-3 and CRF07_BC. The infected primary CD4^+^ cells were subjected to qRT-PCR and immunoblotting analyses. Results indicated that galectin-3 were significantly induced after HIV-1 CRF07_BC and NL4-3 infection (*p* < 0.01) and higher amounts of mRNA and protein of galectin-3 were detected in CRF07_BC than B (*p* < 0.01) (Figure 1E,F). Furthermore, the vector expressing HIV-1 CRF07_BC Tat was transfected into primary CD4^+^ cells via electroporation. Results indicated that CRF07_BC Tat protein significantly induced galectin-3 expression (*p* < 0.01) (Figure 1G). 

### 2.3. Amino Acid Deletions in CRF07_BC p6^Gag^ Ameliorated Galectin-3-Mediated Virus Growth and Budding

**Figure 2:**
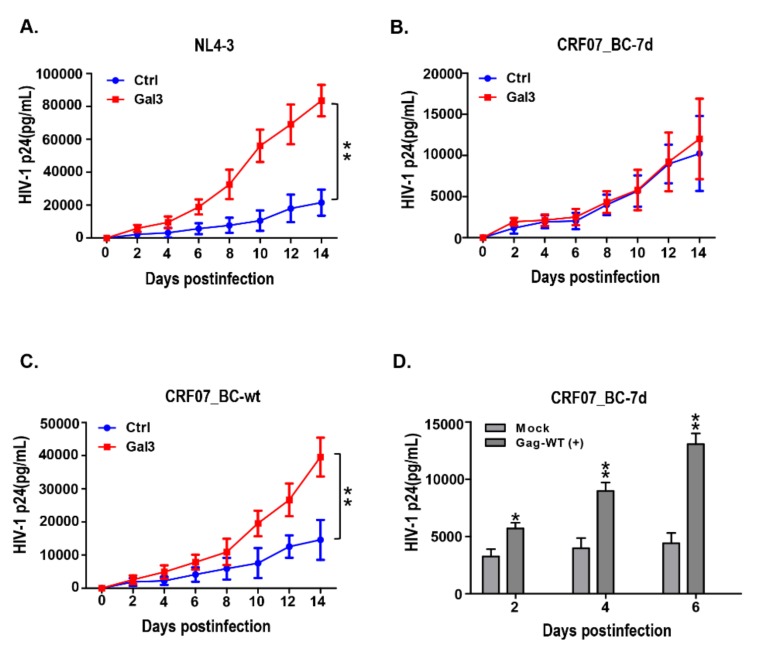
Amino acid deletions in p6^Gag^ reduced galectin-3-mediated promoting effects on viral growth. The control and galectin-3 expressing Jurkat-R5 cells (Jurkat-R5-Ctrl and Jurkat-R5-Gal3 cells) were infected with (**A**) NL4-3, (**B**) CRF07_BC-7d, and (**C**) CRF07_BC-wt. The viral supernatants from these infections were collected for HIV-1 p24 measurements every two days. (**D**) The full-length of Gag expression vector was transfected into Jurkat-R5-Gal3 cells and subjected to CRF07_BC-7d infection. Viral supernatants were collected for HIV-1 p24 determination. Quantitative data represent the means ± SD of results from at least three independent experiments (* *p* < 0.05; ** *p* < 0.01).

Currently, it is still unclear whether deletion of the amino acids in the p6^Gag^ domain of CRF07_BC is involved in the regulation by galectin-3. To address this question, the HIV-1 CRF07_BC infectious clones carrying 7 amino acid deletions and repaired wild-type were generated (hereafter named as CRF07_BC-7d, and CRF07_BC-wt) (Figure S1). These infectious clones were transfected into 239T cells to generate CRF07_BC-7d and CRF07_BC-wt viruses for the following studies. The control and galectin-3 expressing Jurkat-R5 cells (Jurkat-R5-Ctrl and Jurkat-R5-Gal3, respectively) were infected with NL4-3, CRF07_BC-7d, and CRF07_BC-wt viruses and subjected to growth kinetic analysis. Results indicated that galectin-3 expression significantly enhanced NL4-3 growth (Figure 2A). While this effect was not found in CRF07_BC-7d (Figure 2B), galectin-3 expression significantly enhanced CRF07_BC-wt growth (Figure 2C). Further, we noted that enhancement mediated by galectin-3 in CRF07_BC-7d could be significantly recovered when transfected with full-length wild-type Gag (Gag-wt) (*p* < 0.01) (Figure 2D). 

**Figure 3:**
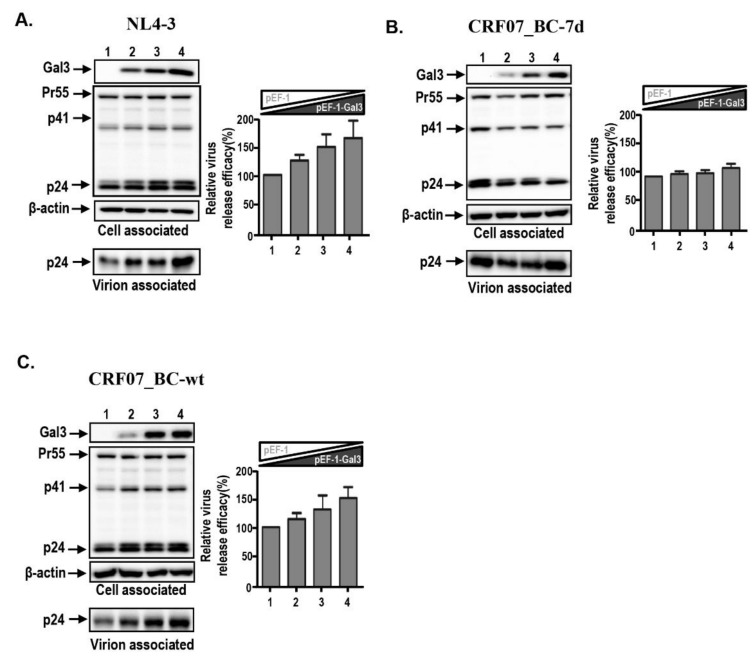
Galectin-3 expression promotes CRF07_BC-wt and NL4-3 viral release. Different ratio of pEF-1 and pEF-1-Gal3 were co-transfected with HIV-1 infectious clones including (**A**) pNL4-3, (**B**) pCRF07_BC-7d, and (**C**) pCRF07_BC-wt into HEK293T cells and incubated at 37 °C for 48 h. HIV-1 p24 determination and immunoblotting analyses were performed by collecting the viral supernatant and cell lysate then exposing with indicated antibodies. By dividing the amount of Gag (p24) in viral lysates by the total amount of Gag (p24) in cell and viral lysates, the relative HIV-1 release efficiency was calculated. Quantitative data represent the means ± SD of results from three independent experiments.

**Figure 4:**
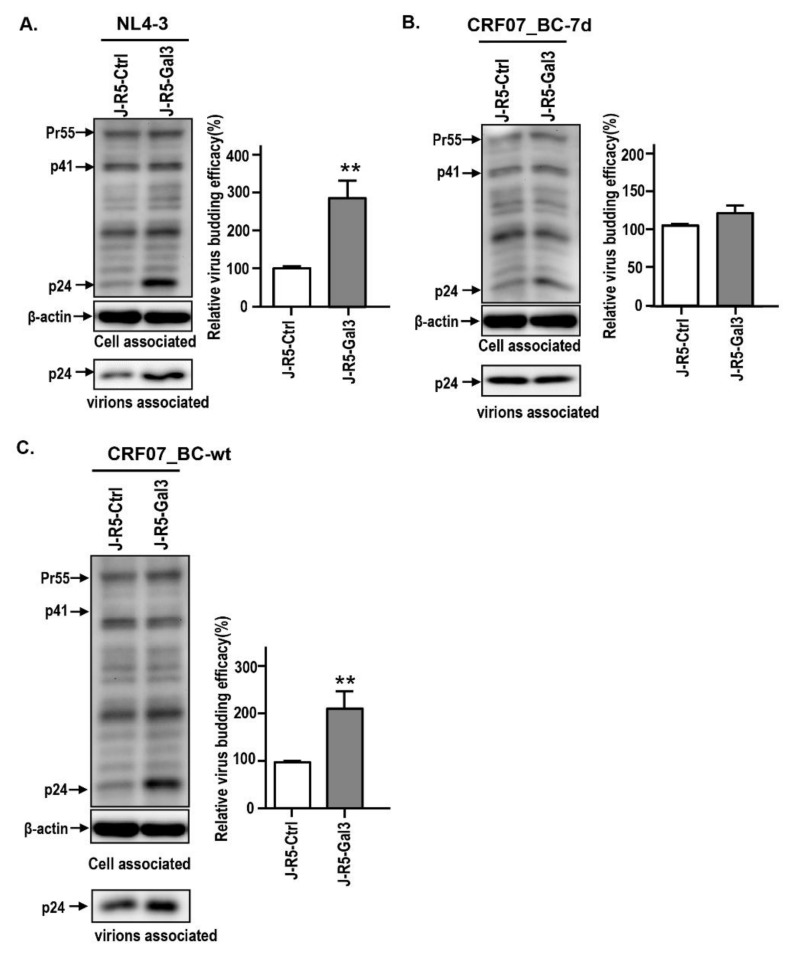
Amino acid deletions in p6^Gag^ reduced galectin-3-mediated CRF07_BC budding. The control and galectin-3 expressing Jurkat-R5 cells were infected with HIV-1 (**A**) pNL4-3, (**B**) pCRF07_BC-7d, and (**C**) pCRF07_BC-wt viruses (multiplicity of infection (MOI) = 0.1). The infected cells were incubated at 37 °C for 48 h. The viral supernatants and cell lysates were collected for immunoblotting analyses with indicated antibodies and HIV-1 p24 determination. The relative HIV-1 release efficiency was calculated by dividing the amount of Gag (p24) in viral lysates by the total amount of Gag (p24) in cell and viral lysates. Quantitative data represent the means ± SD of results from three independent experiments (** *p* < 0.01).

We previously found that galectin-3 positively regulated HIV-1 NL4-3 viral replication kinetics mainly through promotion of viral budding [28]. We therefore hypothesized that galectin-3-regulated CRF07_BC viral growth might affect the viral budding. The viral budding assay was performed using HEK293T cells transfected with different HIV-1 infectious clones combined with measurement of viral supernatants. Results indicated that galectin-3 expression promotes NL4-3 viral budding, instead of CRF07_BC-7d (Figure 3A,B). This galectin-3 promoting effect was improved in CRF07_BC-wt (Figure 3C). Similar phenomena were found in the control and galectin-3 expressing Jurkat-R5 cells, indicating that galectin-3 expression promotes NL4-3 and CRF07_BC-wt viral budding (Figure 4A,C), whereas this effect was not found in CRF07_BC-7d (Figure 4B). Combined, our data indicated that amino acid deletions in p6^Gag^ reduced galectin-3-mediated promotion on CRF07_BC budding, which may correlate with lower viral load and slow progression observed in clinical HIV-1 CRF07_BC infection.

### 2.4. Amino Acids Deletions in CRF07_BC p6^Gag^ Attenuated Galectin-3 Dependent on Alix Association with Gag

**Figure 5:**
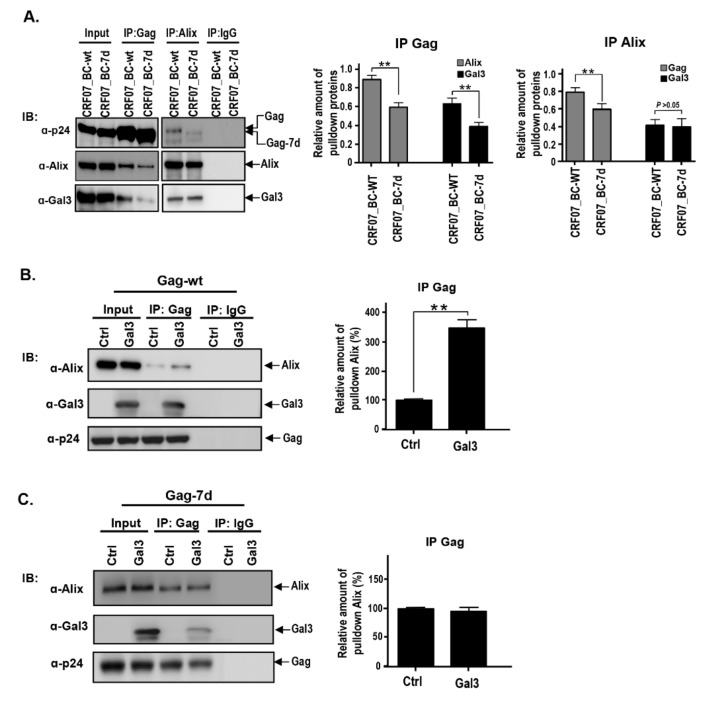
Amino acid deletions in p6^Gag^ attenuated Gag interacting with Alix and galectin-3. (**A**) Magi-5 cells were transfected with pCRF07_BC-7d and pCRF07_BC-wt and incubated at 37 °C for 48 h. The cell lysates were subjected to immunoprecipitation with isotype IgG control, anti-Alix, or anti-Gag antibodies, and immunoblotted with the indicated antibodies. Immunoblot band intensities were quantified by densitometry. Relative amount of pulldown protein was measured using co-immunoprecipitated Alix and Gal3, as well as Gag and Gal3 normalized to pulled down Gag and Alix levels, subsequently. The pEF-1 control and pEF-1-Gal3 were co-transfected with (**B**) pCRF07_BC-7d and (**C**) pCRF07_BC-wt into HEK293T cells and incubated at 37 °C for 48 h. The cell lysates were subjected to immunoprecipitation with isotype IgG control and anti-Gag antibodies and immunoblotted with the indicated antibodies. Densitometry was used to quantify the immunoblot band intensities. Relative amount of pulled down Alix was measured using co-immunoprecipitated Alix normalized to pulled down Gag levels. Representative immunoblotting results are shown. Quantitative data represent the means ± SD of results from three independent experiments (** *p* < 0.01).

The interaction between Alix and Gag has been reported to recruit the ESCRT complex, thus facilitating viral release [16]. Our previous work suggested that galectin-3 expression stabilizes the interaction between Alix and HIV-1 Gag [28]. Our current results indicate that amino acid deletion in the p6^Gag^ leads to a reduction of the galectin-3-mediated promoting effect on CRF07_BC budding. We therefore proposed that amino acid deletion in the p6^Gag^ might reduce the Alix–Gag interaction and that galectin-3 is dependent on Alix to induce the stabilizing effects. The co-immunoprecipitation (Co-IP) assay was performed to test our hypothesis. Magi-5 cells were transfected with pCRF07-BC-wt and pCRF07-BC-7d infectious clones and subjected to the Co-IP assay. Results demonstrated that lower amounts of Alix and galectin-3 were co-precipitated with Gag in CRF07_BC-7d compared to CRF07_BC-wt-transfected groups (Figure 5A). Similarly, lower amounts of Gag co-precipitated with Alix were detected in CRF07_BC-7d compared to CRF07_BC-wt, whereas the non-comparable amount of galectin-3 co-precipitated with Alix in both groups were noted (Figure 5A). Further, control and galectin-3 expressing vectors were co-transfected with CRF07-BC-wt or CRF07-BC-7d infectious clones into HEK293T cells and subjected to the Co-IP assay. Results indicated that expression of galectin-3 led to a significant rise in the levels of Alix co-precipitated with Gag in pCRF07_BC-wt-transfected cells (*p* < 0.05) (Figure 5B), but the galectin-3-mediated enhancement was not noted in the pCRF07_BC-7d-transfected group (*p* > 0.05) (Figure 5C). Combined, these results indicated that galectin-3 was dependent on Alix to interact with Gag, and that amino acid deletions in the p6^Gag^ reduced the galectin-3-mediated promotion of the Alix–Gag association.

## 3. Discussion

In this study, we evaluated the characterization of HIV-1 CRF07_BC from our cohort in Taiwan, as well as the potential role that galectin-3 plays in HIV-1 CRF07_BC infection. Our results demonstrated that CRF07_BC was predominant in HIV-1^+^ IDU groups. Patients who were infected with CRF07_BC had significantly lower viral load and higher CD4 counts compared to the patients infected with HIV-1 B subtype virus. Regarding the role of galectin-3 in CRF07_BC infection, our data indicated that a higher concentration of galectin-3 was detected in the plasma of patients with CRF07_BC infection, CRF07_BC infection upregulated the expression of galectin-3, and the amino acid deletions in CRF07_BC p6^Gag^ ameliorated the galectin-3 regulatory effects. To our knowledge, this is the first study to address the correlation between galectin-3 and CRF07_BC. HIV-1 CRF07_BC has previously caused outbreaks in China and Taiwan and is mainly distributed in the IDU population. Previous studies indicate that HIV-1 CRF07_BC originated in China’s Yunnan Province, with subtype B’ from Thailand mixing with subtype C from India before moving northwestward to Xinjiang Province, along a major Chinese heroin trafficking route, and subsequently disseminating to Taiwan [31,32]. This may have resulted in CRF07_BC becoming the predominant strain circulating in IDUs. Indeed, similar findings were found in other studies indicating that patients infected with CRF07_BC were dominant among IDUs [3,7,8]. Although the mutants with 7–13 amino acid deletion in p6^Gag^ were detected in clinical isolates, the differences among these mutants are not fully addressed. This study found a high percentage of CRF07_BC-7d detected in our cohort. Previous reports concluded that the 7 amino acid deletion is distinctive among virtually all the Taiwanese CRF07_BC strains isolates [6] and mainland China has the deletion in 25.8% of cases [33]. However, such deletions are not detected in HIV-1 subtypes B, C, CRF08_BC, and other BC recombinants. 

The rate of disease progression of HIV-1-infected patients is determined by various factors, such as viral characteristics, immune responses, and host factors. HIV-1 subtype is reported as a major factor correlated with disease progression. Reports indicate that individuals infected with subtypes C, D, and G are more likely to develop AIDS than individuals infected with other subtypes [34,35]. Here, we demonstrated that CRF07_BC infection induced expression and secretion of Gal3, and CRF07_BC-infected individuals displayed slow disease progression. Whether Gal3 induction by HIV-1 subtype B, C, or CRF07_BC correlate with disease progression remains unclear. Furthermore, there was no subtype C patient detected in our cohort, which was a limitation in this study, as we could not determine the characteristics nor the effects of Gal3. We suggest that the role of Gal3 in HIV-1 disease progression among different subtypes is worthy of further investigation. 

HIV-1 CRF07_BC with 7 amino acid deletions covering AIP-1 binding sites occurring naturally was revealed as the predominant strain in our cohort (Table 1). These deletions were also observed in some CRF07_BC isolates from mainland China [36]. Previous studies indicate that phylogenetic analysis using either gag or envelope (Env) gene fragment showed that the Taiwanese CRF07_BC strains were clustered with CRF07_BC strains isolated from Xinjiang (97CN54, 97CN001, and 98CN009) and Guangxi (CNGL-179) [3,5]. However, most of these CRF07_BC isolates from China did not contain amino acid deletions [31,36]. To date, the mechanism of how these deletions are reserved by CRF07_BC virus under serial selection and fitness from the host environment remain unknown. A previous study indicates that a few HIV-1 subtype A strains had 2–5 amino acid deletions in the AIP1-binding domain according to sequence analysis data on HIV-1 strains (including subtype A–D, F–H, J, K, CRF01_AE, and CRF02_AG) [6,37]. Several factors might correlate with HIV-1 fitness, such as replication kinetics, co-receptor usage, N-linked glycosylation sties in Env, transmission route, etc. [38]. Further study is warranted to address these questions.

In addition, whether CRF07_BC containing 7 amino acid deletions has specific preference in the drug resistance profile draws attention. Unfortunately, there were a lack of studies available to address this question. More recently, Hung et al. [39] reported that clinical CRF07_BC-7d isolates with the mutations in the protease (PR) region had obtained the resistance to protease inhibitors (PIs), including ritonavir, saquinavir, indinavir, nelfinavir, and amprenavir (resistant fold range 4.4–47.3). Some studies from mainland China indicate that potential drug-resistant mutants of Chinese CRF07_BC viruses (most isolates were non-amino acid deletion strains) were resistant to Nevirapine (NVP), which is the most frequently used antiretroviral drug in China [40]. However, this drug-resistant phenotype was not observed in Taiwanese CRF07_BC isolates (most isolates containing 7 amino acid deletions). These findings suggest that CRF07_BC with or without amino acid deletions in p6^Gag^ may have different drug resistance preference. 

In recent decades, the importance of glycan and the glycan–lectin interaction have been addressed in many fields. Lectins are carbohydrate-binding proteins, exhibiting high specificity for certain sugar moieties or structures. One of the most known lectins in HIV-1 research is dendritic cell-Specific intercellular adhesion molecule-3-grabbing non-integrin (DC-SIGN), a C-type lectin highly expressed in immature dendritic cells and reported as an alternative receptor to mediate HIV-1 *cis* and *trans* infection. In this study, we found that higher levels of galectin-3 were measured in the plasma from HIV-1-infected patients compared to the healthy control. Among these patients, higher galectin-3 concentrations in CRF07_BC patients was noted. Galectin-3 is known to be highly expressed in many cell types, such as macrophage, dendritic cells (DCs), and T cells. HIV-1 CRF07_BC is an M-tropism virus, propagating mainly in the immune cells expressing CD4^+^ CCR5^+^ co-receptors. We suggested that HIV-1 CRF07_BC infection prompts galectin-3 expression. This may be a result of a part of galectin-3 being secreted out of cells. Additionally, propagation of HIV-1 CRF07_BC would also lead to apoptosis of cells, subsequently causing galectin-3 to be released out. These events might result in the higher level of galectin-3 that was measured in clinical CRF07_BC patients. Furthermore, galectin-3 was reported to be detected in exosomes and exosomes-derived HIV-1-infected cells, such as macrophages or DCs, thus correlating with galectin-3 being detected extracellularly [41].

Our data indicate that clinical HIV-1 CRF07_BC-infected patients have significantly higher serum galectin-3 compared to patients infected with HIV-1 subtype B (Figure 1). We provided evidence that galectin-3 was induced by the HIV-1 Tat protein. Similar regulation was reported by Fogel et al., indicating that expression of Tat protein promotes an increase of galectin-3 in several human cell lines, and the Tat from the B subtype induces a significant upregulation of the 5’-regulatory sequences of the galectin-3 gene [27]. Induction of galectin-3 expression and the galectin-3-dependent Alix-promoting effect on budding step during HIV-1 CRF07_BC infection are two different events, which might occur through different regulatory pathways. However, the details of the mechanisms of these two events remain unclear. Based on our data, we proposed that in the early phase of HIV-1 infection, the Tat could bind to galectin-3 promoter and subsequently trigger cellular galectin-3 expression. These galectin-3 could secrete out upon the non-classical secretion pathway. Regarding the effect of 7 amino acid deletions, we proposed that the negative regulatory effect by amino acid deletions mainly occurs on the late step of the CRF07_BC life cycle. However, whether the 7 amino acid deletion has any effect on galectin-3 secretion and Tat from CRF07_BC or subtype B with different transcription activities, still remain unclear. 

Furthermore, our data demonstrated that 7 amino acid deletion in p6^Gag^ of CRF07_BC significantly ameliorated galectin-3-mediated enhancement in virus budding. We noted that PTAP (Tsg101 binding domain) on the p6^Gag^ region was conserved in all our isolates; however, the 7 amino-acid deletion which truncated the residues overlapped with the Alix-binding domain, especially the residue Y36. Earlier studies determined that Y36A mutation in the p6^Gag^ significantly reduced the interlinkage between Gag and Alix, subsequently ameliorating HIV-1 release [14]. Our previous study indicated that the HIV-1 NL4-3 virus containing Y36A of the p6^Gag^ significantly reduced galectin-3 promotion on viral budding [28]. These data suggested that amino acid deletion in p6^Gag^-ameliorated galectin-3-mediated CRF07_BC budding might be due to the deletion containing an important Alix-binding site, subsequently affecting galectin-3-mediated stabilization on the Alix–Gag interaction. With the exception of Y36A, the potential effect of other deletion residues involved in virus characterization as well as galectin-3-mediated regulation is worthy of further investigation.

In addition, how galectin-3 depends on Alix to express the promoting effect remains an interesting question. The C-terminal region of the p6^Gag^ contains two conserved sequences, PTAP and LYPXL, which interact with Tsg101 and Alix, respectively. These two host cellular proteins initiate a set of sequential interactions leading to the recruitment of members of the endosomal sorting complex required for transport (ESCRT) pathway [14,42]. Furthermore, Alix is comprised of three multiprotein complexes (ESCRT-I, ESCRT-II, and ESCRT-III) that facilitate membrane-modeling events critical for multivesicular body (MVB) generation, cytokinesis, and autophagy [14]. 

Alix is known to consist of three major domains, including the Bro1 domain, the V domain, and a proline-rich region (PRR). The p6^Gag^–Alix interaction is essential during HIV-1 replication and mutations at the Alix-binding site of the p6 results in impaired HIV-1 replication and decreased efficiency of viral release in various cell types [14]. Our results demonstrated that galectin-3 associated with Alix and galectin-3 expression facilitated the Alix–Gag interaction. Nevertheless, this promoting effect could be ameliorated in CRF07_BC with 7 amino acid deletions (Figure 5). Our previous study indicated that the N-terminal region of galectin-3 (a region containing proline-rich tandem repeats) instead of the C-terminal carbohydrate-recognition domain (CRD), binds to Alix [28]. This binding is through protein–protein rather than lectin–glycoconjugate interactions. In addition, based on our data, we suggested that galectin-3 might stabilize the Alix–Gag interaction. Previous studies have reported that some factors (e.g., Tsg101, CEP55, and ALG-2) that bind to the PRR contribute to Alix activation by releasing the PRR from the Bro1-V domain and exposing the YPXL late-domain-binding site [43,44]. Usami et al. indicated that the extreme C-terminus of the PRR is essential for the ability of Alix to promote HIV-1 budding and may connect Alix to a yet-to-be-identified cofactor that is required to support its viral budding function [45]. Based on these findings that galectin-3 binds to the PRR domain of Alix, we suggest that this correlation contributes to Alix activation and to the stable binding of Alix to p6^Gag^.

Galectin-3 and its inhibitors were reported as the potential therapeutic target or antagonist for some diseases [46]. In this study, we proposed that the inhibitors blocking the Gal3–Alix interaction might serve as a potential antiviral drug against HIV-1 infection. Although Alix knockdown greatly reduced HIV-1 release, this knockdown may induce some abnormalities in the treated cells, such as inhibition of actin cytoskeleton assembly, inhibition of ALIX-supported multivesicular body(MVB) sorting, and degradation of activated epidermal growth factor receptor (EGFR) [47]. Another strategy might focus on attenuation of expression or function of galectin-3 via reducing mRNA expression or blocking the N’ or C’ terminal protein–protein interaction. There are some recognized galectin-3 inhibitors, such as TD139 and GR-MD-02, which are proven to have the potential to treat fibrosis and may be considered for the usage of HIV-1 treatment [48].

There were several limitations to this study. The sample size of the study was small and the study participants were not randomly selected; therefore, the study population might not be representative of the general population in Taiwan. For understanding the fine-tune regulatory mechanism, several antiretroviral drugs might be considered as the control to address the role that galectin-3 or the galectin-3–Alix interaction contributes in the late replication or budding of CRF07_BC, such as Bevirimat (BVM), GS-6207, GSK-2838232, and BMS-955176. However, the detailed mechanism of regulation of CRF07_BC by galectin-3 is still not clear; moreover, these drugs are not widely available. There was a limitation to offer some evidence with regard to galectin-3 or galectin-3–Alix in the late replication step by using the HIV-1 maturation inhibitors as a control. 

**Figure 6:**
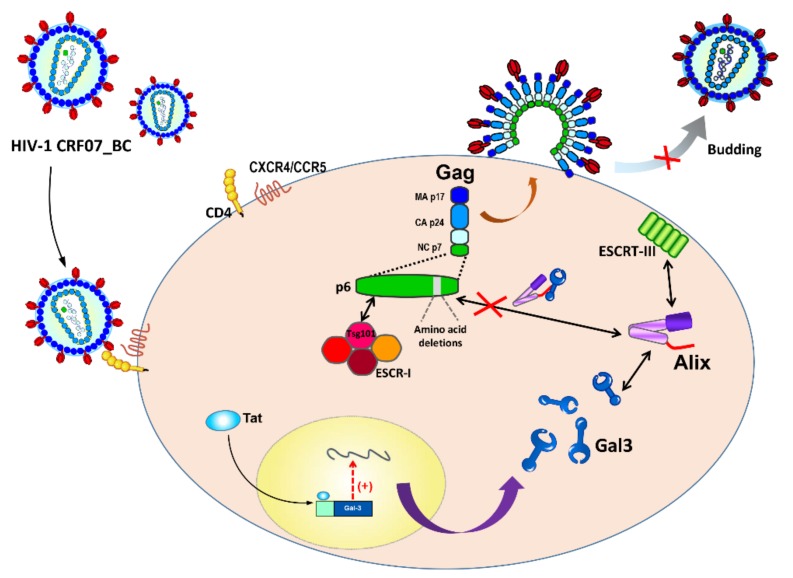
Scheme of how galectin-3 regulates CRF07_BC infection. The red-cross symbol indicates the inhibition of the regulatory process. The single arrow indicates movement of regulatory processes to the following step. The double-sided arrows indicate mutual interaction occurring on both sideds.

In this study, we evaluated the clinical characterization of patients infected with CRF07_BC, virus properties of HIV-1 CRF07_BC, and the effects of amino acid deletions on galectin-3-mediated regulation. Our results suggested that HIV-1 CRF07_BC infection induced galectin-3 expression and amino acid deletion in the p6^Gag^-reduced Alix–Gag interaction, subsequently ameliorating virus budding (Figure 6). This study concludes that the intact Alix-binding site on the p6^Gag^ is essential for galectin-3-mediated regulation on budding of HIV-1 CRF07_BC, and that galectin-3 has potential as an alternative antiviral target. 

## 4. Materials and Methods 

### 4.1. Ethics Statement

All study participants provided a written informed consent form. Approval was applied for and received from the Institutional Ethics Committee of the Kaohsiung Medical University, Taiwan (KMUHIRB-SV(II)-20160066, 28 December 2016). All procedures were performed in accordance to committee guidelines. 

### 4.2. Cell and Viruses 

This research utilized various cell lines including HEK293T cells (ATTC No. CRL-3216), Jurkat T cells (ATCC No. TIB-152), and Magi-5 cells (HeLa cells expressing CD4, CXCR4, and CCR5, and containing the β-galactosidase gene controlled by HIV-1 LTR), which were obtained through the NIH

AIDS Reagent Program. Human primary CD4^+^ T cells were isolated from peripheral blood mononuclear cells of healthy adult HIV-1(−) donors. Human primary CD4^+^ T cells were purified by negative selection using a magnetic-activated cell sorting system (Miltenyi Biotec, Bergisch-Gladbach, Germany) following the protocols from elsewhere [28]. HIV-1 B subtype NL4-3 viruses were generated by transfecting pNL4-3 in HEK293T cells while CRF07_BC viruses were obtained by clinical isolation or transfecting the infectious clones generated in this study. HIV-1 virions were purified by sucrose gradients according to previously described procedures [49,50]. 

### 4.3. Plasmids 

The NL4-3 infectious clone was obtained from the US NIH AIDS Research and Reference Reagent program. The CRF07_BC infectious clones were generated in this study via cloning the full-length sequence of clinical CRF07_BC patients to the pUC-18 vector. The detail of construction of CFR07_BC infectious clone was described elsewhere. pET-Gal3 and pcDNA3 Flag-Tat was offered by Dr. Fu-Tong Liu at Institute of Biomedical Sciences, Academia Sinica, Taiwan. 

### 4.4. Determination of HIV-1 Genotypes

The HIV-1 genotypes were determined following a previous publication [51]. Briefly, the blood samples were drawn from the enrolled patients. The PBMCs were purified from whole blood using Ficoll-Paque^TM^ density gradient centrifugation. The proviral DNA was extracted using QIAamp DNA Blood Mini Kit (QIAGEN, Stanford, CA, USA). The gag-gene regions were amplified by PCR. Nested multiplex PCR was performed to determine HIV-1 subtypes. The genotype-specific primers were referenced by a previous HIV-1 genotyping assay based on different sizes of amplification PCR products corresponding to each different subtype of HIV-1 [51]. Once multiplex PCR showed two or more HIV-1 subtypes suggesting dual infection, serial PCR using a single pair of subtype-specific primers was used for confirmation of dual infection.

### 4.5. Viral Growth Kinetic Assay

Direct infection assays were performed utilizing Jurkat-R5-control and Jurkat-R5-Gal3 cells (1 × 10^5^/well). Briefly, HIV-1 NL4-3 or CRF07_BC viruses (MOI = 0.1–0.01) were subjected to incubation with cells for 2 h at 37 °C in serum-free medium, followed by rinsing in phosphate-Buffered Saline (PBS). Subsequently, 2 mL of Roswell Park Memorial Institute Medium (RPMI) or Dulbecco’s Modified Eagle Medium (DMEM) medium containing 10% serum, antibiotics, and polybrene (8 μg/mL) were added, followed by incubation in 5% CO_2_ at 37 °C. The 100 μL supernatants were collected and refilled with 100 μL fresh culture medium in each well every two days. Collected viral supernatants were subjected to HIV-1 p24 quantification using Alliance HIV-1 P24 Antigen ELISA Kit (PerkinElmer; Cat. No. NEK050B, Waltham, MA, USA). 

### 4.6. Immunoblotting

Analyzed proteins were quantified and used for immunoblotting analyses. The equal quantified proteins or equal volumes of immunoprecipitated proteins were analyzed by SDS-PAGE. Separated proteins were transferred to nitrocellulose membranes (PolyScreen, PerkinElmer). Transferred antigens were incubated with the following antibodies: mouse anti-galectin-3, rabbit anti-Alix, mouse anti-HIV-1 p24 (Millipore, Burlington, MA, USA), mouse anti-β-actin (Sigma, St. Louis, MO, USA), or rabbit anti-α-tubulin (Epitomics, Burlingame, CA, USA), and held for 1 h at 37 °C. After three washes with 1x phosphate-buffered saline with 0.1% Tween^®^ detergent (PBST), membranes were incubated with horseradish peroxidase (HRP)-conjugated antibodies (goat anti-mouse IgG, goat anti-human IgG, or goat anti-rabbit IgG) (Amersham Biosciences, Charlemagne, UK) for 1 h at 37 °C. Hybridized protein bands were created with an Immobilon™ Western Enhanced Chemiluminescence (ECL) protein detection system (Millipore, Burlington, MA, USA).

### 4.7. Co-Immunoprecipitation 

The details of the co-immunoprecipitation assay were described previously [28]. Briefly, HEK293T cells were transfected with plasmids (pEF1-Ctrl, pEF1-Gal3, pNL4-3, pCRF_07BC-7d, pCRF_07BC-wt) using Effectene (QIAGEN), and then incubated for 48 h at 37 °C. After three washes with lactose (30 mM) to remove extracellular and un-crosslinked galectins, cells were treated with 8 μg/mL of the crosslinker DSP (3,3’-dithiodipropionic acid (N-hydroxysuccinimide ester)) (SIGMA) at room temperature for 30 min. This was followed by adding one-tenth volume of 1 M Tris-HCl (pH 7.5) and incubation at room temperature for 15 min. The cells were then lysed in NP-40 buffer (150 mM NaCl, 25 mM Tris-HCl (pH 7.4), 1 mM EDTA, 0.5% NP-40, and 5% glycerol containing protease inhibitor cocktail) (CALBIOCHEM, Fort Kenner, NJ, USA) and the lysates were centrifuged at 16,000× *g*. Supernatants were immunoprecipitated with anti-Alix or anti-p24. Aliquots were analyzed by SDS-PAGE and immunoblotting, using antibodies against Alix, galectin-3, and HIV-1 p24. 

### 4.8. qRT-PCR 

For measuring Gal3 mRNA expression, the qRT-PCR was performed. The protocol was published elsewhere [52]. Briefly, the primary human CD4^+^ T cells were purified from peripheral blood mononuclear cells (PBMCs) by negative selection using the magnetic-activated cell sorting (MACS) system (Miltenyi Biotec) and then subjected to be incubated with HIV-1 CRF07_BC viruses (MOI = 0.1). After incubation for 48 h, the mock control and infected cells were subjected to total RNA extraction using the EasyPrep Total RNA Kit (BIOTOOLS, Cat No. DPT-BD19) and reverse transcribed using TOOLs Easy Fast RT Kit (BIOTOOLS, Cat No. KRT-BA18, New Taipei City, Taiwan), according to the manufacturer’s protocol. Real-time PCR analysis of Gal3 mRNA expression was performed using TOOLS Easy SYBR qPCR Mix kit (BIOTOOLS, Cat No. FPT-BB01-4) and an ABI 7500 thermocycler. Primers used in qRT-PCR were as follows: human Gal-3-F (5′-GGCCACTGATTGTGCCTTAT-3′) and Gal3-R (5′-TCTTTCTTCCCTTCCCCAGT-3′). Results were normalized to glyceraldehyde-3-phosphate dehydrogenase using the comparative threshold cycle method.

### 4.9. Statistical Analysis

All experiments were performed at least three times each. SAS statistic software (SAS version 9.1; SAS Institute, Cary, CA, USA) or GraphPad Prism software was used for statistical significance, and the level of significance was set at *p* < 0.05.

## Figures and Tables

**Table 1:** Characteristics of the study population.

	HIV-1-Infected Population		
Characteristics	Male (%) (*n* = 32)	Female (%) (*n* = 6)	Total (%) (*n* = 38)
**Age (years)**			
15–29	14(43.8)	2(33.3)	16(42.1)
30–49	16(50)	3(50)	19(50)
≥50	2(6.2)	1(16.7)	3(7.9)
**Viral Load (copies/mL)**			
<10,000	2(6.2)	1(16.7)	3(7.8)
10,000–100,000	19(59.4)	3(50)	22(57.9)
>100,000	11(34.4)	2(33.3)	13(34.2)
**CD4 Count (cells/mm^3^)**			
<200	5(15.6)	1(16.7)	6(15.8)
200–500	21(65.6)	4(66.7)	25(65.8)
>500	6(18.8)	1(16.7)	7(18.4)
**Serum galectin-3 (mean ± SD)**	3.49 ± 0.51	3.24 ± 0.27	3.45 ± 0,49
**HIV-1 subtype**			
CRF01_AE	2(6.3)	3(50)	5(13.2)
B	7(21.9)	1(16.7)	8(22.2)
C	0	0	0
CRF07_BC	23(71.9)	2(33.3)	25(69.4)
CRF08_BC	0	0	0

**Table 2:** HIV-1 genotyping of our recruited HIV-1(+) subjects.

	HIV-1 CRF07_BC Population		
Virus Subtype	Male (%) (*n* = 23)	Female (%) (*n* = 2)	Total (%) (*n* = 25)
**CRF07_BC**			
Gag-7d	21(91.3)	2(100)	23(92)
Gag-11d	2(8.7)	0	2(8)
Gag-13d	0	0	0
Gag-wt	0	0	0

## Supplementary Materials

Supplementary materials can be found at https://www.mdpi.com/1422-0067/21/8/2910/s1.

## Abbreviations

Gal3	Galectin-3
Alix	ALG-2 interacting protein X
CRF07_BC-7d	HIV-1 CRF07_BC virus with 7 amino acid deletions in p6^Gag^
CRF07_BC-wt	HIV-1 CRF07_BC virus non-deletion wild-type

## References

[1] Su L., Graf M., Zhang Y., von Briesen H., Xing H., Kostler J., Melzl H., Wolf H., Shao Y., Wagner R. (2000). Characterization of a virtually full-length human immunodeficiency virus type 1 genome of a prevalent intersubtype (C/B’) recombinant strain in China. J. Virol..

[2] Yang R., Xia X., Kusagawa S., Zhang C., Ben K., Takebe Y. (2002). On-going generation of multiple forms of HIV-1 intersubtype recombinants in the Yunnan Province of China. AIDS.

[3] Chen Y.M., Lan Y.C., Lai S.F., Yang J.Y., Tsai S.F., Kuo S.H. (2006). HIV-1 CRF07_BC infections, injecting drug users, Taiwan. Emerg. Infect. Dis..

[4] Chang S.Y., Sheng W.H., Lee C.N., Sun H.Y., Kao C.L., Chang S.F., Liu W.C., Yang J.Y., Wong W.W., Hung C.C. (2006). Molecular epidemiology of HIV type 1 subtypes in Taiwan: outbreak of HIV type 1 CRF07_BC infection in intravenous drug users. AIDS Res. Hum. Retrovir..

[5] Lin H.H., Shih Y.L., Liu Y.C., Lee S.S., Huang C.K., Chen Y.L., Chin C., Lai C.H., Tsai H.C., Guo Y.C. (2006). An epidemic of HIV type I CRF07_BC infection among injection drug users in Taiwan. J. Acquir. Immune. Defic. Syndr..

[6] Lin Y.T., Lan Y.C., Chen Y.J., Huang Y.H., Lee C.M., Liu T.T., Wong W.W., Yang J.Y., Wang C.T., Chen Y.M. (2007). Molecular epidemiology of HIV-1 infection and full-length genomic analysis of circulating recombinant form 07_BC strains from injection drug users in Taiwan. J. Infect. Dis..

[7] Huang S.W., Wang S.F., Lin Y.T., Yen C.H., Lee C.H., Wong W.W., Tsai H.C., Yang C.J., Hu B.S., Lin Y.H. (2014). Patients infected with CRF07_BC have significantly lower viral loads than patients with HIV-1 subtype B: mechanism and impact on disease progression. PLoS ONE.

[8] Lin P.H., Lai C.C., Yang J.L., Huang H.L., Huang M.S., Tsai M.S., Yang C.J., Cheng C.L., Su Y.C., Chang S.F. (2013). Slow immunological progression in HIV-1 CRF07_BC-infected injecting drug users. Emerg. Microbes. Infect..

[9] Weiss E.R., Gottlinger H. (2011). The role of cellular factors in promoting HIV budding. J. Mol. Biol..

[10] Martin-Serrano J., Neil S.J. (2011). Host factors involved in retroviral budding and release. Nat. Rev. Microbiol..

[11] Jacks T., Power M.D., Masiarz F.R., Luciw P.A., Barr P.J., Varmus H.E. (1988). Characterization of ribosomal frameshifting in HIV-1 gag-pol expression. Nature.

[12] Partin K., Krausslich H.G., Ehrlich L., Wimmer E., Carter C. (1990). Mutational analysis of a native substrate of the human immunodeficiency virus type 1 proteinase. J. Virol..

[13] Hurley J.H., Emr S.D. (2006). The ESCRT complexes: structure and mechanism of a membrane-trafficking network. Annu. Rev. Biophys. Biomol. Struct..

[14] Fujii K., Munshi U.M., Ablan S.D., Demirov D.G., Soheilian F., Nagashima K., Stephen A.G., Fisher R.J., Freed E.O. (2009). Functional role of Alix in HIV-1 replication. Virology.

[15] Hu H.L., Meng Z.F., Zhang X.Y., Lu J.X. (2011). [HIV-1 infection affects the expression of host cell factor TSG101 and Alix]. Bing Du Xue Bao.

[16] Fujii K., Hurley J.H., Freed E.O. (2007). Beyond Tsg101: the role of Alix in ‘ESCRTing’ HIV-1. Nat. Rev. Microbiol..

[17] Liu F.T., Bevins C.L. (2010). A sweet target for innate immunity. Nat. Med..

[18] Vasta G.R. (2009). Roles of galectins in infection. Nat. Rev. Microbiol..

[19] Diaz-Alvarez L., Ortega E. (2017). The Many Roles of Galectin-3, a Multifaceted Molecule, in Innate Immune Responses against Pathogens. Mediat. Inflamm..

[20] Thery C., Boussac M., Veron P., Ricciardi-Castagnoli P., Raposo G., Garin J., Amigorena S. (2001). Proteomic analysis of dendritic cell-derived exosomes: a secreted subcellular compartment distinct from apoptotic vesicles. J. Immunol..

[21] Yang M.L., Chen Y.H., Wang S.W., Huang Y.J., Leu C.H., Yeh N.C., Chu C.Y., Lin C.C., Shieh G.S., Chen Y.L. (2011). Galectin-1 binds to influenza virus and ameliorates influenza virus pathogenesis. J. Virol..

[22] Shim J.A., Park S., Lee E.S., Niki T., Hirashima M., Sohn S. (2011). Galectin-9 ameliorates herpes simplex virus-induced inflammation through apoptosis. Immunobiology.

[23] Garner O.B., Aguilar H.C., Fulcher J.A., Levroney E.L., Harrison R., Wright L., Robinson L.R., Aspericueta V., Panico M., Haslam S.M. (2010). Endothelial galectin-1 binds to specific glycans on nipah virus fusion protein and inhibits maturation, mobility, and function to block syncytia formation. PLoS Pathog..

[24] Yang R.Y., Rabinovich G.A., Liu F.T. (2008). Galectins: Structure, function and therapeutic potential. Expert Rev. Mol. Med..

[25] Chen H.Y., Fermin A., Vardhana S., Weng I.C., Lo K.F., Chang E.Y., Maverakis E., Yang R.Y., Hsu D.K., Dustin M.L. (2009). Galectin-3 negatively regulates TCR-mediated CD4+ T-cell activation at the immunological synapse. Proc. Natl. Acad. Sci. USA.

[26] Wang W.H., Lin C.Y., Chang M.R., Urbina A.N., Assavalapsakul W., Thitithanyanont A., Chen Y.H., Liu F.T., Wang S.F. (2019). The role of galectins in virus infection—A systemic literature review. J. Microbiol. Immunol. Infect..

[27] Fogel S., Guittaut M., Legrand A., Monsigny M., Hebert E. (1999). The tat protein of HIV-1 induces galectin-3 expression. Glycobiology.

[28] Wang S.F., Tsao C.H., Lin Y.T., Hsu D.K., Chiang M.L., Lo C.H., Chien F.C., Chen P., Arthur Chen Y.M., Chen H.Y. (2014). Galectin-3 promotes HIV-1 budding via association with Alix and Gag p6. Glycobiology.

[29] Hsu D.K., Hammes S.R., Kuwabara I., Greene W.C., Liu F.T. (1996). Human T lymphotropic virus-I infection of human T lymphocytes induces expression of the beta-galactoside-binding lectin, galectin-3. Am. J. Pathol..

[30] Fang Z., Xing H., Meng Z., Hong K., Liao L., He X., Shao Y. (2010). Genetic characterization analysis of the tat exon-1 region of HIV type 1 CRF07_BC strains in China. AIDS Res. Hum. Retrovir..

[31] Su L., Zhou X., Yuan D., Yang H., Wei D., Qin G., Liang S. (2014). Prevalence and patterns of drug-resistance mutations among HIV-1 patients infected with CRF07_BC strains in Sichuan province, China. Virol. Sin..

[32] Wang Z., Hong K., Zhang J., Zhang L., Li D., Ren L., Liang H., Shao Y. (2013). Construction and characterization of highly infectious full-length molecular clones of a HIV-1 CRF07_BC isolate from Xinjiang, China. PLoS ONE.

[33] Song Y.H., Meng Z.F., Xing H., Ruan Y.H., Li X.P., Xin R.L., Ma P.F., Peng H., Shao Y. (2007). Analysis of HIV-1 CRF07_BC gag p6 sequences indicating novel deletions in the central region of p6. Arch. Virol..

[34] Kanki P.J., Hamel D.J., Sankale J.L., Hsieh C., Thior I., Barin F., Woodcock S.A., Gueye-Ndiaye A., Zhang E., Montano M. (1999). Human immunodeficiency virus type 1 subtypes differ in disease progression. J. Infect. Dis..

[35] Coetzer M., Cilliers T., Papathanasopoulos M., Ramjee G., Karim S.A., Williamson C., Morris L. (2007). Longitudinal analysis of HIV type 1 subtype C envelope sequences from South Africa. AIDS Res. Hum. Retrovir..

[36] Wu Y., Wang H., Ren X., Wan Z., Hu G., Tang S. (2017). HIV-1 CRF07_BC with a Seven Amino Acid Deletion in the gag p6 Region Dominates in HIV-1-Infected Men Who Have Sex with Men in China. AIDS Res. Hum. Retrovir..

[37] Marlowe N., Flys T., Hackett J., Schumaker M., Jackson J.B., Eshleman S.H., Network H.I.V.P.T., Adult A.C.T.G. (2004). Analysis of insertions and deletions in the gag p6 region of diverse HIV type 1 strains. AIDS Res. Hum. Retrovir..

[38] Biesinger T., Kimata J.T. (2008). HIV-1 Transmission, Replication Fitness and Disease Progression. Virology.

[39] Huang S.W., Li W.Y., Wang W.H., Lin Y.T., Chou C.H., Chen M., Huang H.D., Chen Y.H., Lu P.L., Wang S.F. (2017). Characterization of the Drug Resistance Profiles of Patients Infected with CRF07_BC Using Phenotypic Assay and Ultra-Deep Pyrosequencing. PLoS ONE.

[40] Wu H., Zhang H.J., Zhang X.M., Xu H.F., Wang M., Huang J.D., Zheng B.J. (2012). Identification of drug resistant mutations in HIV-1 CRF07_BC variants selected by nevirapine in vitro. PLoS ONE.

[41] Kulkarni R., Prasad A. (2017). Exosomes Derived from HIV-1 Infected DCs Mediate Viral trans-Infection via Fibronectin and Galectin-3. Sci. Rep..

[42] Garrus J.E., von Schwedler U.K., Pornillos O.W., Morham S.G., Zavitz K.H., Wang H.E., Wettstein D.A., Stray K.M., Cote M., Rich R.L. (2001). Tsg101 and the vacuolar protein sorting pathway are essential for HIV-1 budding. Cell.

[43] Carlton J.G., Agromayor M., Martin-Serrano J. (2008). Differential requirements for Alix and ESCRT-III in cytokinesis and HIV-1 release. Proc. Natl. Acad. Sci. USA.

[44] Zhai Q., Landesman M.B., Chung H.Y., Dierkers A., Jeffries C.M., Trewhella J., Hill C.P., Sundquist W.I. (2011). Activation of the retroviral budding factor ALIX. J. Virol..

[45] Usami Y., Popov S., Gottlinger H.G. (2007). Potent rescue of human immunodeficiency virus type 1 late domain mutants by ALIX/AIP1 depends on its CHMP4 binding site. J. Virol..

[46] Sciacchitano S., Lavra L., Morgante A., Ulivieri A., Magi F., De Francesco G.P., Bellotti C., Salehi L.B., Ricci A. (2018). Galectin-3: One Molecule for an Alphabet of Diseases, from A to Z. Int. J. Mol. Sci..

[47] Sun S., Zhou X., Corvera J., Gallick G.E., Lin S.H., Kuang J. (2015). ALG-2 activates the MVB sorting function of ALIX through relieving its intramolecular interaction. Cell Discov..

[48] Chan Y.C., Lin H.Y., Tu Z., Kuo Y.H., Hsu S.D., Lin C.H. (2018). Dissecting the Structure-Activity Relationship of Galectin-Ligand Interactions. Int. J. Mol. Sci..

[49] Villard R., Hammache D., Delapierre G., Fotiadu F., Buono G., Fantini J. (2002). Asymmetric synthesis of water-soluble analogues of galactosylceramide, an HIV-1 receptor: new tools to study virus-glycolipid interactions. Chembiochem.

[50] Lund N., Branch D.R., Mylvaganam M., Chark D., Ma X.Z., Sakac D., Binnington B., Fantini J., Puri A., Blumenthal R. (2006). A novel soluble mimic of the glycolipid, globotriaosyl ceramide inhibits HIV infection. AIDS.

[51] Wei M., Guan Q., Liang H., Chen J., Chen Z., Hei F., Feng Y., Hong K., Huang H., Xing H. (2004). Simple subtyping assay for human immunodeficiency virus type 1 subtypes B, C, CRF01-AE, CRF07-BC, and CRF08-BC. J. Clin. Microbiol..

[52] Hayashi Y., Jia W., Kidoya H., Muramatsu F., Tsukada Y., Takakura N. (2019). Galectin-3 Inhibits Cancer Metastasis by Negatively Regulating Integrin beta3 Expression. Am. J. Pathol..

