# Supplementary material for: Amino Acid Deletions in p6Gag Domain of HIV-1 CRF07_BC Ameliorate Galectin-3 Mediated Enhancement in Viral Budding
Source: Int J Mol Sci. 2020 Apr 21;21(8):2910. doi: 10.3390/ijms21082910 (PMC7216183; doi:10.3390/ijms21082910)
Supplement: Supplementary file 1 [file ijms-21-02910-s001.pdf]

Supplementary Materials

Materials and methods:

Construction of a full-length CRF07_BC molecular clone

A full-length CRF07_BC molecular clone was constructed with the strategy published elsewhere. Briefly, a full-length CRF07_BC-p6Gag-wt HIV-1 genome was constructed by ligation of wildtype HIV CRF07_BC PCR product into pUC57 plasmid with EcoRV and SphI restriction enzyme. The pCRF07_BC-p6Gag-7d and the pCRF07_BC-p6Gag-11d plasmid was derived from pCRF07_BC with a deletion of 7 amino acids in p6 (30PIDKELY36) and 11 amino acids in p6 (26QKQEPIDKELY36) by site-directed mutagenesis respectively.

Results:

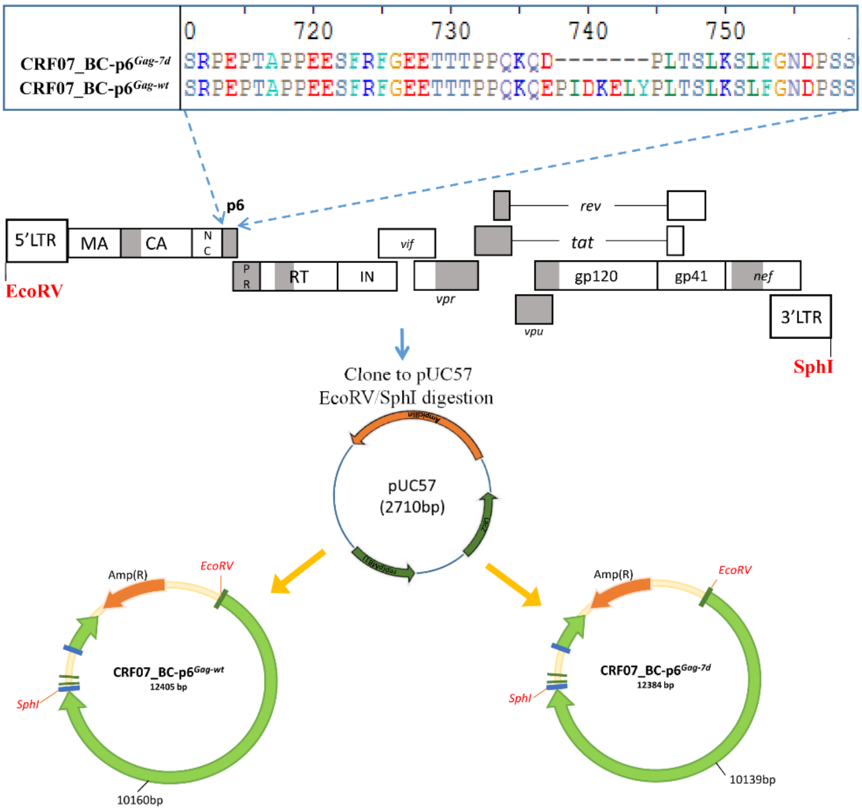

Figure S1. Construction of CRF07_BC infectious clones. The full-length of CRF07_BC with non-deletion wild-type was cloned to pUC57. Further, the 7-11 amino acid deletions were generated via site-directed mutagenesis.
